# Relationship between anaemia, hypoalbuminaemia, and dietary lifestyle of the older adults attending a primary care clinic in Nigeria

**DOI:** 10.4314/gmj.v57i4.7

**Published:** 2023-12

**Authors:** Abdulgafar L Olawumi, Bukar A Grema, Abdullahi K Suleiman, Godpower C Michael, Zainab A Umar, Abiso A Mohammed, Ahmad I Rufai, Mahmud B Mahmud, Hauwa A Muhammad

**Affiliations:** 1 Aminu Kano Teaching Hospital - Family Medicine, Kano, Nigeria; 2 University of Maiduguri Teaching Hospital - Family Medicine, Maiduguri, Borno, Nigeria; 3 Aminu Kano Teaching Hospital – Haematology and Blood Transfusion, Kano, Nigeria

**Keywords:** Anaemia, Hypoalbuminaemia, Dietary Lifestyle, Older Patients, Primary Care

## Abstract

**Objectives:**

To determine the prevalence and severity of anaemia and assess the relationship between dietary lifestyle, hypoalbuminaemia, and anaemia of older persons

**Design:**

A cross-sectional hospital-based study.

**Setting:**

This study was conducted in the General Outpatient Clinic, the primary care unit of Aminu Kano Teaching Hospital in Kano, Nigeria.

**Participants:**

A total of 378 patients aged ≥ 60 years who presented to the General Out-patient Clinic.

**Main outcome measures:**

Prevalence and severity of anaemia, relationship between anaemia and hypoalbuminaemia, and dietary lifestyle of the participants.

**Results:**

A total of 348 respondents completed the study. The mean age of respondents was 67.83 ±7.53 years, with female (60.9%) predominance. The prevalence of anaemia and hypoalbuminaemia were 42.2% and 17.8%, respectively. Hypoalbuminaemia (β=0.335, 95%CI=0.131-0.229, *P*<0.001), long duration of comorbidities (β= -0.179, 95%CI= -0.165-0.047, *P*<0.001), one full meal/day (β=0.130, 95%CI=0.224-1.879, *P*=0.013), and low monthly income (β=0.122, 95%CI=0.179-1.543, *P*=0.026) were the predictors of anaemia among the older persons in this study.

**Conclusion:**

This study revealed a high prevalence of anaemia among older adults. The identified predictors, such as hypoalbuminaemia, long duration of comorbidities, reduced food intake and low monthly income, will be useful in developing guidelines and strategies for managing the condition in primary care settings and other similar sites.

**Funding:**

None declared.

## Introduction

Anaemia is a common medical condition in the elderly and has been identified as one of the factors contributing to their health decline.^1^ It has been linked to a variety of medical problems, such as an increased number of falls, loss of functional independence, cognitive alterations and depression, with an overall increase in morbidity and mortality among older persons.^2^ The haematocrit, PCV or haemoglobin (Hb) concentration, as well as the red blood cell (RBC) count, are commonly used to define anaemia.^3^ The World Health Organization (WHO) defines anaemia as having a haemoglobin concentration of less than 13 g/dl in men and less than 12 g/dl in women.^4^ Although there are some controversies regarding whether these levels should be used to identify anaemia in older persons, there is no commonly accepted alternative definition of anaemia in this age group.^5^,^6^

In general, older adults have lower haemoglobin levels than younger persons.^5^ The reasons for this are unclear. However, it has been suggested to be a component of natural ageing, and the presence of co-morbidities might worsen the anaemia.^6^

Anaemia is more common as people get older, and the prevalence is higher among men and black populations.^7^ A systematic review by Gaskell *et al* reported an overall mean prevalence of 17% for anaemia among older adults in developed countries, with 12% in community-dwelling older adults, 40% in hospital admissions, and 47% in nursing home residents.^6^ However, higher prevalence was reported in developing countries, ranging from 31.1% in hospital-based studies to 47.7% in community-based studies and as high as 68.7% in nursing homes.^8^,^9^,^10^

The socio-demographic and lifestyle differences might be responsible for the difference in the prevalence of anaemia between developed and developing countries.

The causes of anaemia in the elderly can be broadly classified under three main categories: nutritional deficiency (iron, folate or vitamin B12), anaemia of inflammation or of chronic disease, and unexplained anaemia.^11^ These three causes of anaemia have also been linked with low albumin blood level (hypoalbuminaemia), which was acknowledged to be a significant risk factor for mortality and morbidity in older persons.^12^ Anaemia and hypoalbuminaemia in older people have been shown to be associated with frailty, which also has a close relationship with their impaired nutritional status.^12^

The results of previous studies indicated that inadequate dietary intake, poor nutritional status, and heavy smoking or alcohol consumption were correlated with iron metabolism or the risk of anaemia.^8^,^9^,^13^ As a result, the dietary lifestyle pattern is now viewed as a novel strategy to be used in nutritional epidemiology to evaluate disease risk.^13^

Every country in the world is experiencing growth in both the size and the proportion of older persons in the population.^14^ Hence, most countries are preparing for these major challenges to ensure that their health and social systems are ready to manage and benefit from this demographic transformation.^14^ Anaemia, which is an essential factor in health decline, has been extensively studied in Nigerian children, women of reproductive age, and pregnant women, and it has been discovered to be high and largely due to dietary factors.^15^-^17^ However, data on anaemia and associated factors in the elderly from developing countries, especially Nigeria, is scarce.^18^ Also, to our knowledge, there are no published studies on anaemia among the elderly population in northern Nigeria. Hence, the aim of this study is to determine the prevalence and severity of anaemia and evaluate the relationship between anaemia, hypoalbuminaemia, and the dietary lifestyle of older persons.

## Methods

The study was a cross-sectional design, conducted in a General Outpatient Clinic (GOPC), which is the primary care unit of Aminu Kano Teaching Hospital (AKTH) in Kano. The hospital is a 750-bed health facility which serves about 13 million population in Kano and its environment. The hospital medical record revealed that older persons account for about 15% (38) of the 250 patients seen daily in the GOPC. The elderly male and female patients aged 60 years and above who presented at the clinic within the 12-week study period (18th April 2022 to 10th July, 2022), constituted the study population. Consenting elderly patients attending the hospital's GOPC who had not been transfused with red blood cells within the previous three months were recruited. Critically ill elderly patients or those with major neuropsychiatric illnesses were exempted from the study since they might not comply with the study procedures.

### Sample Size

A total of 378 was estimated as the sample size using the formula:^19^ n= Zα^2^pq/d^2^ where; n = minimum sample size, Zα = standard normal deviate corresponding to a 5% level of significance (1.96), P = (54.5%, prevalence of anaemia among elderly in a similar setting in Ethiopia),^20^ since there was no published hospital-based study in Nigeria.

q = 1-p (45.5%), the proportion of the elderly who are not anaemic.

d = level of precision which was set as 5%.

The finite correction formula^19^ n_s_ = n / 1+ (n/N) was used to adjust the sample size to 378, using a projected response rate of 90%.

### Sampling Method

A systematic sampling technique was used to recruit 378 elderly patients attending the hospital, using the sampling frame of 3,192 (38x7x12) and a sample interval of 8 (3192/378). The first respondent was selected via balloting on the first day, and then every eighth elderly patient who met the inclusion criteria was recruited.

### Data Collection

The questionnaire was administered to the participants after pre-testing on the older patients attending the GOPC of Murtala Muhammed Specialist Hospital (MMSH) in Kano. To avoid duplication, the folders of respondents were serialised and given numbers. When necessary, information was confirmed using the patient's medical records or the carers—the questionnaire comprised sections which included socio-demographic characteristics, dietary history, medical history and laboratory parameters. The dietary lifestyle questionnaire (history of full meal/day, meat, fish or legumes/day, fruits or vegetables/day, fluid intake/day, need for assistance during feeding, difficulty in swallowing, and self-view of nutritional Status) was adapted from the dietary component of the mini-nutritional assessment tool for the older persons.^21^ The face and content validity were assessed by the pre-tested older patients and nutritional experts, respectively. A Cronbach alpha of 0.830 was obtained for internal consistency. This indicates the high dependability of the tool. The self-view of nutritional status is the subjective evaluation of one's own level of nutrition.

Each patient's packed cell volume (PCV) was measured using the anti-coagulant-coated haematocrit tube. After filling the tube to 2/3rd level with the patient's blood, spun by the centrifuge machine and then read by the haematocrit reader, the PCV was recorded and graded according to the WHO grading of anaemia: normal (≥36%), mild (30-35%), moderate (21-29%) and severe anaemia (<21%).^22^ About 2mls of venous blood was also taken with a 2ml syringe and 21G hypodermic needle under aseptic condition. It was collected into a lithium heparin bottle and transported to the AKTH chemical pathology laboratory to determine the serum albumin levels. A value ≥ 35g/L was taken as normal, while < 35g/L was taken to be low.^21^ Sample collection for PCV and serum albumin, including analysis and reporting, were supervised by one of the researchers who is a laboratory scientist.

### Statistical analysis

Data were collated and analysed by using the IBM SPSS (Statistical Package for Social Sciences) version 21 software. Data were cross-checked for completeness, entered into an Excel sheet and cleaned before importing into SPSS. Descriptive analyses of all variables were performed to identify ambiguous and missing values. The chi-square test and Pearson correlation coefficient were used to assess the significance of associations between categorical variables (such as gender, tribe and dietary lifestyle) and continuous variables (such as age, monthly income, serum albumin and duration of comorbidities), respectively. Variables with p ≤ 0.05 at the bivariate level were entered into a multiple linear regression model to identify the predictors of anaemia among the respondents.

### Ethical considerations

Ethical approval was obtained from the Research Ethical Committee of the Hospital (No. NHREC/28/01/2020/AKTH/EC/3273). The date of approval was 13/04/2022. Participants discovered to have anaemia, hypoalbuminaemia or poor dietary lifestyle during the study were provided with adequate counselling and care as appropriate.

## Results

A total of 348 respondents completed the study to give a 92% response rate. Their ages ranged from 60 to 95 years, with a mean age of 67.83 ±7.53 years. The majority (78.2%) of the respondents were 60-74 years old. They were predominantly females (60.9%) and Hausa tribe (58.9%). Most of them were either married (50.0%) or widowed (46.0%), belonging to an extended family structure (87.1%). The majority (73.6%) had no formal education, and (57.5%) earned below N30,000 (50 USD) per month (Table 1).

**Table 1:** Socio-demographic characteristics of the respondents (n=348)

Variables	Frequency n (%)
**Age group (years)**	
**60-74**	272 (78.2)
**75-84**	61 (17.5)
**≥ 85**	15 (4.3)
**Mean ± SD = 67.83 ± 7.53**	
**Sex**	
**Male**	136 (39.1)
**Female**	212 (60.9)
**Marital status**	
**Married**	174 (50.0)
**Divorced/separated**	14 (4.0)
**Widowed**	160 (46.0)
**Tribe**	
**Hausa**	205 (58.9)
**Fulani**	102 (29.3)
*Others	41 (11.8)
**Educational status**	
**None**	256 (73.6)
**Primary**	31 (8.9)
**Secondary**	16 (4.6)
**Tertiary**	45 (12.9)
**Occupation**	
**Civil servant**	22 (6.3)
**Self-employed**	109 (31.3)
**Unemployed**	177 (50.9)
**Retired**	40 (11.5)
**Monthly income**	
**< 50 USD**	200 (57.5)
**50-99 USD**	70 (20.1)
**100-149 USD**	38 (10.9)
**≥ 150 USD**	40 (11.5)
**Median (IQR) = 31.67(53.33) USD**	
**Living arrangement**	
**Alone**	4 (1.1)
**Nuclear family**	41 (11.8)
**Extended family**	303 (87.1)

*Other tribe: Yoruba, Igbo, Ebira, Edo, Idoma and Kanuri * IQR: Interquartile rang

As shown in Table 2, the majority (69.5%) of the respondents took three full meals daily and could feed without assistance (70.7%); however, a significant proportion of them (58.3%) experienced difficulty with swallowing. The majority of the respondents (65.2%) took a certain portion of vegetables/fruits every day; however, most of them were still uncertain (70.9%) about their self-assessment of their nutritional status, which could not be unconnected to their low intake of fluid (41.4%) and proteinous foods (44.0%) such as meat, fish or legumes. The most prevalent comorbidity among the respondents was hypertension (50.0%), followed by arthritis (23.7%), diabetes mellitus (15.3%) and poor vision (5.0%). Most (74.4%) of the respondents had been diagnosed with the comorbidities for over 5 years.

**Table 2:** Dietary lifestyle of the respondents (n=348)

Variables	Frequency n (%)
**Full meal/day**	
**1**	7 (2.0)
**2**	99 (28.5)
**3**	242 (69.5)
**Needs assistance during feeding**	
**Every time**	3 (0.9)
**Sometimes**	99 (28.4)
**None**	246 (70.7)
**Difficulty in swallowing**	
**Yes**	203 (58.3)
**No**	145 (41.7)
**Meat or fish or legumes/day**	
**Yes**	153 (44.0)
**No**	195 (56.0)
**Fruit or vegetables/day**	
**Yes**	121 (34.8)
**No**	227 (65.2)
	
**< 3 cups**	204 (58.6)
**3 to 5 cups**	115 (33.1)
**> 5 cups**	29 (8.3)
**Self-View of Nutritional Status**	
**Malnourish**	18 (5.2)
**Normal**	83 (23.9)
**Uncertain**	247 (70.9)

As shown in Table 3, the mean PCV of the respondents was 35.16 ± 4.23%. About 58% of the respondents had normal PCV (≥ 36%), while 33.6% and 8.6% had mild (30-35%) and moderate (21-29%) anaemia respectively. However, none of the participants had severe anaemia (< 21%).

**Table 3:** Anaemia and other clinical indices of the respondents (n=348)

Variables	Frequency n (%)
**Anaemia classified by PCV**	
**No anaemia (≥36%)**	201 (57.8)
**Mild anaemia (30-35%)**	117 (33.8)
**Moderate anaemia (21-29%)**	30 (8.6)
**Mean ± SD = 35.16 ± 4.23%**	
**Duration of co-morbidities**	
**< 5 years**	88 (25.3)
**5 – 10 years**	126 (36.2)
**> 10 years**	134 (38.5)
**Mean ± SD = 11.58 ± 7.00**	
**Serum albumin**	
**Normal**	286 (82.2)
**Low**	62 (17.8)
**Mean ± SD = 42.72 ± 7.73**	
**Packed cell volume (PCV)**	
**Normal (≥36%)**	201 (57.8)
**Anaemia (< 36%)**	147 (42.2)
**Mean ± SD = 35.16 ± 4.23**	

Hence, the prevalence of anaemia in this study was 42.2%, with female preponderance. The mean value of serum albumin of the study respondents was 42.72 ± 14.57g/L. The majority (82.2%) had normal serum albumin levels (≥35g/L), while 17.8% had low (< 35g/L) levels.

Table 4 showed statistically significant associations between anaemia and advancing age (χ^2^= 22.105, p < 0.001), lower educational level (χ^2^= 15.828, p < 0.001), occupation (χ^2^= 8.327, p = 0.040), and low monthly income (χ^2^= 8.384, p = 0.039).

**Table 4:** Socio-demographic characteristics and anaemia (n =348)

Variables	No Anaemia	Anaemia	χ^2^	P value
**Age group**			22.105	< 0.001*
**60-74**	175(64.3%)	97(35.7%)		
**75-84**	21(34.4%)	40(65.6%)		
**≥ 85**	5(33.3%)	10(66.7%)		
**Sex**			0.104	0.747
**Male**	80(58.8%)	56(41.2%)		
**Female**	121(57.1%)	91(42.9%)		
**Marital status**				0.097
**Married**	108(62.1%)	66(37.9%)		
**Divorced/separate**	10(71.4%)	4(28.6%)		
**Widowed**	83(51.9%)	77(48.1%)		
**Tribe**			3.802	0.149
**Hausa**	118(57.6%)	87(42.4%)		
**Fulani**	54(52.9%)	48(47.1%)		
**Others**	29(70.7%)	12(29.3%)		
**Educational status**			15.828	0.001*
**None**	134(52.3%)	122(47.7%)		
**Primary**	23(74.2%)	8(25.8%)		
**Secondary**	15(93.8%)	1(6.2%)		
**Tertiary**	29(64.4%)	16(35.6%)		
**Occupation**			8.327	0.040*
**Civil servant**	15(68.2%)	7(31.8%)		
**Self-employed**	71(65.1%)	38(34.9%)		
**Unemployed**	89(50.3%)	88(49.7%)		
**Retired**	26(65.0%)	14(35.0%)		
**Monthly income**			8.384	0.039*
**< 50 USD**	106(53.0%)	94(47.0%)		
**50-99 USD**	41(48.6%)	29(41.4%)		
**100-149 USD**	23(60.5%)	15(39.5%)		
**≥ 150 USD**	31(77.5%)	9(22.5%)		
**Living arrangement**			3.726**	0.149
**Alone**	3(75.0%)	1(25.0%)		
**Nuclear family**	29(70.7%)	12(29.3%)		
**Extended family**	169(55.8%)	134(44.2%)		

* Statistically significant

** Fishers exact

Table 5 showed a statistically significant association between anaemia and dietary lifestyle practices of the respondents such as one full meal/day (FE= 18.432, P < 0.001), need of assistance during feeding (FE= 14.448, P = 0.001), difficulty in swallowing (χ^2^= 19.871, p < 0.001), daily intake of meat, fish or legume (χ^2^= 7.625, p = 0.006) and self-view of nutritional status (χ^2^= 7.898, p = 0.019). A similar significant association was also found between anaemia and serum albumin (χ^2^= 26.614, p < 0.001) and long duration of comorbidities (χ^2^= 13.955, p = 0.001). However, none of the comorbidities had a significant association with anaemia.

**Table 5:** Dietary lifestyle and anaemia (n =348)

Variables	No Anaemia	Anaemia	χ^2^	P value
**Full meal/day**			18.432**	<0.001*
**1**	1(14.3%)	6(85.7%)		
**2**	43(43.4%)	56(56.6%)		
**3**	157(64.9%)	85(35.1%)		
**Needs assistancing during feeding**			14.448**	0.001*
**Every time**	1(33.3%)	2(66.7%)		
**Sometimes**	42(42.4%)	57(57.6%)		
**None**	158(64.2%)	88(35.8%)		
**Difficulty in swallowing**			19.871	<0.001*
**Yes**	97(47.8%)	106(52.2%)		
**No**	104(71.7%)	41(28.3%)		
**Meat or fish or legumes/day**			7.625	0.006*
**Yes**	101(66.0%)	52(34.0%)		
**No**	100(51.3%)	95(48.7%)		
**Fruits or vegetables/day**			0.064	0.80
**Yes**	71(58.7%)	50(41.3%)		
**No**	130(57.3%)	97(42.7%)		
**Fluid intake/day**			1.491	0.475
**< 3 cups**	122(59.8%)	82(40.2%)		
**3 to 5 cups**	65(56.5%)	50(43.5%)		
**> 5 cups**	14(48.3%)	15(51.7%)		
**Self-View of Nuritional Status**			7.898	0.019*
**Malnourish**	5(27.8%)	13(72.2%)		
**Uncertain**	143(57.9%)	104(42.1%)		
**Normal**	53(63.9%)	30(36.1%)		

* Statistically significant

** Fisher's Exact Test

Further assessment of the correlation between packed cell volume and continuous variables still revealed a significant association and a positive correlation with albumin (r = 0.393, p < 0.001) and monthly income (r = 0.244, p < 0.001), but a negative correlation with age (r = -0.281, p < 0.001) and duration of co-morbidities (r = -0.226, p < 0.001).

As depicted in Table 6, a multiple regression analysis was run to predict hypoalbuminaemia (β=0.335, 95%CI=0.131-0.229, *P*<0.001), long duration of comorbidities (β= -0.179, 95%CI=-0.165-0.047, *P*<0.001), one full meal/day (β=0.130, 95%CI=0.224-1.879, *P*=0.013), and low monthly income (β=0.122, 95%CI=0.179-1.543, *P*=0.026) as independent determinant of anaemia among the older persons in this study.

**Table 6:** Predictors of anaemia among the respondents (n =348)

Variables	β	95% CI	p-value
**Age**	-0.089	-0.111 – 0.015	0.132
**Educational status**	0.078	-0.101 – 0.716	0.140
**Occupation**	-0.013	-0.578 – 0.440	0.789
**Low monthly income**	0.122	0.179 – 1.543	0.026*
**Needs assistance during feeding**	-0.005	-0.974 – 1.063	0.931
**Difficulty in swallowing**	0.034	-0.566 – 1.136	0.511
**One full meal/day**	0.130	0.224 – 1.879	0.013*
**Low intake of meat or fish or legumes/day**	0.039	-0.485 – 1.128	0.433
**Self-View of malnutrition**	0.078	-0.235 – 1.508	0.152
**Long duration of co-morbidities**	-0.179	-0.165 – 0.047	<0.001*
**Hypoalbuminaemia**	0.335	0.131 – 0.229	<0.001*

* Statistically significant, β: Standardized coefficients, CI: Confidence interval

## Discussion

This study is one of the few in Nigeria that provide data on anaemia in the older persons. That was why its was discussed at the 2023 West African College of Physicians AGSM. The study aimed to determine the prevalence and severity of anaemia, and evaluate the relationship between anaemia, hypoalbuminaemia, and dietary lifestyle of the older persons.

The prevalence of anaemia among the elderly in this study was 42.2%; with majority (33.6%) having mild anaemia and few (8.6%) having moderate anaemia while none had severe anaemia. This is closer to 41% reported by Ferreira *et al* among institutionalized elderly in Brazil, and 45.2% reported by Orces *et al* in Ecuador.^23^,^24^ The fmding is also comparable to the overall prevalence of anaemia among older people in nursing homes and hospitals, which was shown to be 47% and 40%, respectively, in a systematic review by Gaskell *et al.*^6^ The high prevalence of anaemia in this study and other similar hospital or institution-based studies could be due to high prevalence of co-morbidities associated with the aged population.^20^,^25^ According to a prospective cohort analysis of 3,758 patients aged 65 and above, both new-onset anemia and low hemoglobin levels were linked to higher morbidity and mortality.^26^ The most common cause of anaemia identified among the older population was anaemia of chronic diseases as a result of prolonged drug intake, feeding and swallowing problems and consequently malnutrition.^26^ These were also the typical findings in this study. A higher prevalence of 54.5% was reported for anaemia among elderly outpatients in Ethiopia,^20^ and 53% among cohort of hospitalised elderly in France.^27^ The lower prevalence of anaemia reported in the index study as compared to Melku et al study in Ethiopia may be explained by high proportion of vegans among their respondents.^20^ Since, anaemia worsens with advancing age,^5^-^10^ the higher prevalence of anaemia reported among respondents in France could be due to the high age cutoff of 65 years as compared 60 years used in this study.^27^ However, a lower prevalence of 21.1% was reported by Bach *et al* in a large cohort study in European University Hospitals.^1^ Similar lower prevalence were reported by Oldewage-Theron *et al* in a peri-urban settlement in South Africa (25.7%),^28^ Vadakattu *et al* among urban elderly in India (20.6%),^29^ and overall prevalence in older adults (17%) by Gaskell *et al* with more steep among the community-dwelling older adults (7-11%).^6^

These variations in the prevalence of anaemia in different countries could be due to different study designs, lifestyle, geographical variations, and different age cutoffs (60 years or 65 years) in various studies.

Among the dietary lifestyle practices of the respondents, this study reported a high level of difficulty in swallowing (58.3%), low daily intake of meat, fish or legumes (56%) and fluid intake (58.6%). Also, about 5% viewed themselves as malnourished, and 71% were uncertain about their nutritional status. The high prevalence of swallowing difficulty and other nutritional problems is similar to other findings.^30^-^32^ This was attributed to the decline in appetite, swallowing and food consumption with increasing age (physiological anorexia), and it worsens with progressive functional impairment and chronic morbidities.^33^,^34^

Although age was not a predictor of anaemia in this study, older age was associated with anaemia. Various studies also revealed a similar finding.^1^-^3^,^5^-^10^,^31^-^34^ As people age, their erythroid precursors in their bone marrow diminish, and they become less receptive to stimulatory growth factors.^32^ Additional reasons found in this study include; swallowing difficulties, a low-protein diet, insufficient fluid intake, loss of appetite, and a negative self-perception of one's nutritional state as one ages.

The strong relationship between anaemia and low income or financial dependency is consistent with other studies.^10^,^33^,^34^ This might be because financial dependence affects people's purchasing power, calories available per person, and access to healthcare services. Similarly, due to competition for limited resources, feeding the older persons in the family may not be adequately prioritised. This will indirectly affect their nutritional status and, consequently, their risk of anaemia. This is evidenced by the high prevalence of anaemia among the elderly living in an extended family setting in this study.

This study revealed that taking only one full meal per day is a predictor of anaemia among the elderly. Some studies also reported the association between anaemia and reduced dietary/calorie intake frequency.^26^,^28^,^29^,^31^ This is not surprising because anaemia is an important consequence of poor nutrition.^32^ The concept of “physiological anorexia makes the elderly less hungry and fuller before meals and as a result, elderly individuals eat more slowly and consume smaller meals”.^30^,^31^ This could be worse with reduced frequency of dietary intake coupled with the background underlying illnesses that cause increased nutrient requirements, increased nutrient loss or poor nutrient absorption.^30^

There was a significant association but weak negative correlation between anaemia and long duration of comorbidities among the elderly. This findings was similar to other studies.^6^,^8^ This was connected to an increase in the probability of chronic drug use, recurrent hospital visits, and, subsequently, a higher likelihood of hospitalisation or institutionalisation.^8^ There was also a significant association and moderate positive correlation between anaemia and hypoalbuminaemia among the elderly in this study. This is consistent with the findings of the few studies that assess the relationship between anaemia and hypoalbuminaemia in the elderly.^12^,^35^ A longitudinal population-based observational survey by Corona *et al* in Brazil reported a significant association between anaemia and hypoalbuminaemia. They concluded that anaemia and hypoalbuminaemia are important markers for death in older adults and have an additive effect on mortality.^12^ A retrospective study by Röhrig *et al* on 626 German elderly patients found a significant association between anaemia and hypoalbuminaemia.^35^ They also reported a moderate positive correlation between anaemia and hypoalbuminaemia, and they concluded that anaemic patients are at increased risk of hypoalbuminaemia, and this risk increases even more in malnourished patients.^35^ Although the exact reason for this association is unknown, albumin is a transport protein for folate and vitamin B12, and lack of albumin might result in increased levels of free folate and vitamin B12, leading to anaemia.^36^

One of the strengths of our study is that all participants were selected using the random sampling method. Also, this study contributes to fill the gap in the dearth of literature on anaemia prevalence in older adults. We could not determine the causes of anaemia in this population because mean corpuscular volume (MCV), mean corpuscular haemoglobin (MCH), Vitamin B12, folate, and iron levels were not assessed. This is a limitation of our study. However, despite these limitations, the data from this study will be useful in developing guidelines for assessing risk factors, evaluating anaemia in older patients, and managing the condition in primary care settings and other places or sites with similar characteristics. Future studies should assess other anaemic parameters, such as MCV, MCH, vitamin B12, folate, and iron levels, in order to classify and determine the causes of anaemia in this special population.

## Conclusion

This study revealed a high prevalence of anaemia among older adults. The identified predictors, such as hypoalbuminaemia, long duration of comorbidities, reduced food intake and low monthly income, will be useful in developing guidelines and strategies for the management of anaemia among older persons in the primary care settings and other similar sites.
